# Reviewing the Diagnostic Performance of 99mTc-TRODAT-1 Imaging in Distinguishing Idiopathic Parkinson's Disease from Parkinson-Plus Syndromes

**DOI:** 10.1055/s-0044-1787995

**Published:** 2024-07-04

**Authors:** Tejasvini Singhal, Manishi L. Narayan, Rajat Manchanda, Parneet Singh, Minakshi Dhar, Ashutosh Tiwari, Niraj Kumar

**Affiliations:** 1 Department of Nuclear Medicine, All India Institute of Medical Sciences, Bhubaneswar, Bhubaneswar, Orissa, India; 2 Department of Nuclear Medicine, All India Institute of Medical Sciences, Rishikesh, Rishikesh, Uttarakhand, India; 3 Department of Neurology, Neuro Care Hospital, Karnal, Haryana, India; 4 Department of Internal Medicine, All India Institute of Medical Sciences, Rishikesh, Rishikesh, Uttarakhand, India; 5 Department of Neurology, All India Institute of Medical Sciences, Rishikesh, Rishikesh, Uttarakhand, India

**Keywords:** 99mTc-TRODAT-1, idiopathic Parkinson's disease, Parkinson-plus syndrome, movement disorders, parkinsonism

## Abstract

**Aim** Diagnosing movement disorders can be challenging owing to their similar clinical presentations with other neurodegenerative and basal ganglia disorders, like idiopathic Parkinson's disease (IPD), essential tremors (ET), vascular parkinsonism, multiple system atrophy (MSA), and progressive supranuclear palsy (PSP). Technetium-99m labeled tropane derivative (99mTc-TRODAT-1) imaging can help in diagnosing Parkinson's disease at an early stage to help early initiation of the treatment. The current study aimed to evaluate the role of 99mTc-TRODAT-1 imaging in differentiating IPD and Parkinson-plus syndromes (PPS).

**Material and Methods** We have analyzed 38 patients, referred to our department for 99mTc-TRODAT imaging. These patients were thoroughly evaluated in the movement disorder clinic at our institute and had a possible/ probable diagnosis of IPD, Hoehn and Yahr (H&Y) stage I/II ( *n* = 28) or PPS (PSP [ *n* = 06] and MSA [ *n* = 04]). Striatal uptake ratio (SUR) was calculated in all the patients and data was statistically analyzed.

**Results** The mean age of IPD, PSP, and MSA groups was 56.5 ± 12.15, 65.2 ± 11.1, and 51.2 ± 3.9 years, respectively. On qualitative evaluation, all patients had reduced striatal uptake on 99mTc-TRODAT imaging, with 31/38 patients showed a greater reduction in putaminal uptake compared with the caudate nucleus. On semiquantitative evaluation, mean total SUR was 0.58 ± 0.27, 0.53 ± 0.31, and 0.91 ± 0.20 in IPD, PSP, and MSA groups, respectively. The total SUR was lowest in the PSP group followed by IPD, but MSA had relatively higher SUR, although the difference was not statistically significant. Among the IPD patient group, 25/28 patients (89.3%) experienced a greater reduction in SUR values in the striatum contralateral to the side, where motor symptoms first manifested at disease onset.

**Conclusion** 99mTc-TRODAT is a potential imaging biomarker for the evaluation of presynaptic dopaminergic dysfunction in patients with movement disorders. In our study cohort, mean SUR values were lowest for the PSP group followed by IPD and MSA group, which was in concordance with previous studies. However, the difference between SUR values in these two groups was not statistically significant. The present study emphasizes that the capacity of 99mTc-TRODAT-1 imaging alone for diagnosing IPD from PPS is constrained, although it offers a precise approach for distinguishing patients with IPD from those with essential tremors, drug-induced, or psychogenic parkinsonism. Consequently, more specific imaging biomarkers are needed to effectively differentiate between patients with IPD and those with PPS.

## Introduction

Dopamine is an endogenous catecholamine with various neuronal (as neurotransmitter) and nonneuronal actions (as autocrine or paracrine agent). It plays a crucial role in the control of movement by modulating neuronal activity in the substantia nigra pars compacta/ventral tegmental area. Dopaminergic system dysfunction is associated with multiple neurodegenerative disorders. Parkinson's disease (PD) is the most common neurodegenerative disorder associated with dopaminergic system dysfunction. It is more common in old age with a prevalence of 0.1 to 0.2% in general population, 1% in the age group of 65 to 70 years, and 3% in individuals above 80 years of age. 1 Idiopathic PD (IPD) is primarily caused by insufficiency in the striatal dopaminergic system, which results from the loss of dopaminergic neurons in the substantia nigra. This deficiency leads to the characteristic motor symptoms of IPD, which include rigidity, tremors, akinesia, and changes in speech and gait. The current diagnosis of IPD is substantially based on the clinical symptoms as well as a favorable response to levodopa therapy. 2 There is no significant role of anatomic imaging modalities such as magnetic resonance imaging (MRI) and computed tomography (CT) in either diagnosing PD or in monitoring the disease-related changes over the course of time. 3

Parkinson-plus syndrome (PPS) are a heterogeneous group of movement disorder encompassing multiple system atrophy (MSA), progressive supranuclear palsy (PSP), and corticobasal degeneration. These demonstrate distinct clinical features in addition to classical parkinsonian features, poor response to dopamine, and are generally associated with poorer prognosis, thus making the accurate diagnosis important. However, it is difficult to distinguish these from IPD especially in early stages. 4

PSP presents with features of parkinsonism along with slow ocular saccades, eyelid apraxia, and restricted eye movements with impairment of downward gaze. 5 MSA is an adult-onset, sporadic, progressive neurodegenerative disease characterized by varying severity of parkinsonian features, cerebellar ataxia, autonomic failure, urogenital dysfunction, and corticospinal disorders. 6

Correct and early diagnosis of PD and its differentiation from other movement disorders especially PPS is necessary for early administration of targeted therapy, counseling, and prognostication. 3

Nuclear medicine imaging with technetium-99m labeled tropane derivative (99mTc-TRODAT-1) can pick the neurobiological abnormalities well before the anatomical abnormality. 99mTc-TRODAT-1 binds to dopamine transporter (DAT) present on the presynaptic dopamine neuron terminals and regulates the synaptic dopamine levels by reuptake mechanism. 7 DAT is considered to be a marker for the functional integrity of dopamine neurons. Thus, functional imaging with 99mTc-TRODAT-1 can help in early and accurate diagnosis as well as in individualizing treatment. Positron emission tomography (PET) has established its role in the evaluation of motor disorders but is limited by its availability. Therefore, there is a need to evaluate single-photon emission CT (SPECT)-based imaging biomarkers like 99mTc-TRODAT-1 in the evaluation of movement disorders. The current study evaluated the role of 99mTc-TRODAT-1 in the evaluation of IPD and its differentiation from PPS.

## Material and Methods

This was a prospective study conducted after obtaining approval from the Institutional Ethics Board (IEC No. IEC/20/416). The study included patients > 18 years of age, willing to give informed consent to undergo the study with a possible/probable diagnosis of IPD, and referred for 99mTc-TRODAT-1 imaging from the movement disorder clinic. Patients in remission, diagnosed with secondary parkinsonism (drug-induced parkinsonism and vascular parkinsonism), unable to cooperate with SPECT/CT imaging, and pregnant and lactating females were excluded from the study.

All patients underwent 99mTc-TRODAT-1 imaging after intravenous injection of 666 to 740 MBq of Tc-99m-TRODAT-1. The tracer was administered following meticulous radiolabeling and routine quality control checks. SPECT/CT images of brain were acquired 4 hours after injection using a dual-head camera GE-NMCT 670, SPECT-CT (GE Healthcare).

**Fig. 1:**
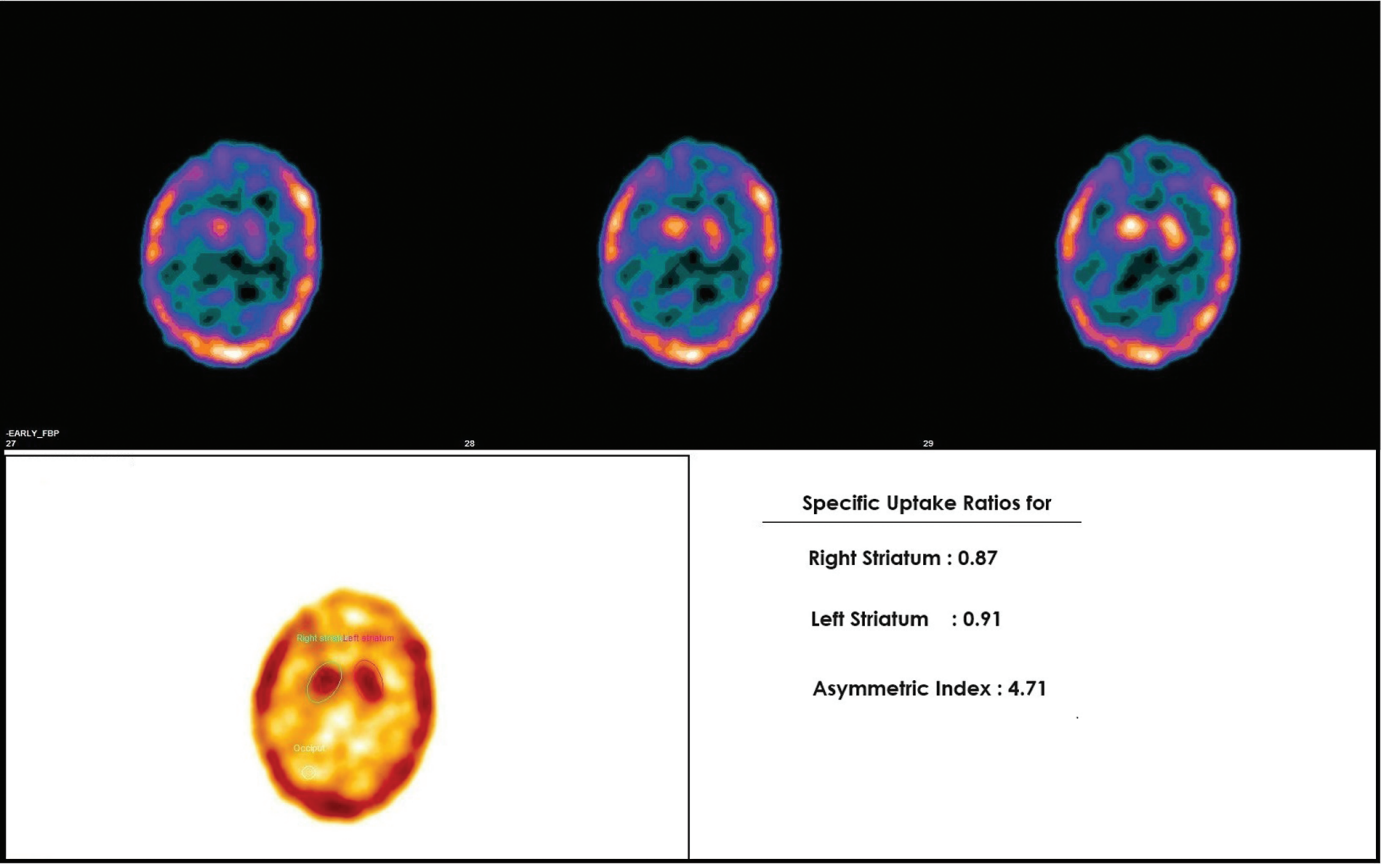
Technetium-99m labeled tropane derivative (99mTc-TRODAT-1) brain single-photon emission computed tomography (SPECT) imaging for a patient with essential tremors. Showing semiquantitative evaluation. Region of interests (ROIs) were drawn over bilateral striatum and occipital cortex as the background. Striatal uptake ratios (SURs) were calculated as striatal ROI average counts per pixel (ACP) — occipital ROI ACP/striatal ROI ACP.

Acquired images were transferred to a dedicated Xeleris workstation for processing. SPECT images were interpreted by an experienced nuclear medicine physician. The SPECT images were reconstructed in axial, coronal, and sagittal planes and the reconstructed images were coregistered with corresponding CT image of each patient along the canthomeatal line. Reconstructed and coregistered SPECT/CT images were subjected to both qualitative and semiquantitative analyses. Visual analysis of the 99mTc-TRODAT uptake in bilateral striatum was done and asymmetry of tracer uptake on each striatum, if present, was also noted. For semiquantitative analysis, transverse slices were selected. Regions of interest (ROIs) were drawn for the striatum of each side on composite images of five consecutive transverse slices, which showed the highest basal ganglia activity. Occipital cortex ROI was drawn which served as the background tracer activity and the average counts per pixel (ACP) were calculated ( Fig. 1 ). The striatal uptake ratio (SUR) of each striatum were calculated which is



Asymmetric index was calculated as



where ILSBR is the ipsilateral striatal binding ratio and CLSBR is the contralateral striatal binding ratio.

The patients were divided into IPD, PSP, and MSA groups based on clinical follow-up/management response. SUR and asymmetric index (AI) were evaluated and compared between these groups. Also, Hoehn and Yahr (H&Y) stage was evaluated in patients with IPD and correlated with the imaging features.

### Statistical Analysis

Data was analyzed using SPSS version 23.0 for Windows OS. A *p* -value of < 0.05 was considered significant between the different groups. The values for numerical data were expressed as mean ± standard deviation and categorical data was expressed as proportions. Normality of the data was checked with Shapiro–Wilk test, using SPSS. The data was analyzed using analysis of variance test. The correlation coefficient ( *r* -value) was measured to analyze the relationship between clinical parameters and SUR in IPD patients.

## Results

A total of 42 participants were referred for 99mTc-TRODAT imaging from the department of neurology. Four participants from the group of parkinsonism were excluded from the study for various reasons; imaging could not be completed in one patient due to claustrophobia and 3/42 were found to have alternate diagnosis (essential tremors, drug-induced Parkinson's and dystonia, and exhibited normal 99mTc-TRODAT SUR). A total of 38 eligible patients with clinical diagnosis of parkinsonism (IPD, PPS) who underwent 99mTc-TRODAT imaging satisfactorily at our institute, were analyzed. Demographic details of these patients are given in Table 1 .

**Table 1:** Demographic and clinical characteristics of the study population

Demographic and clinical characteristics	Idiopathic Parkinson's disease ( *n* = 28)	Parkinson-plus syndrome ( *n* = 10)	
		PSP ( *n* = 06)	MSA ( *n* = 04)
Age at inclusion in the study (y)	56.5 ± 12.15	65.2 ± 11.1	51.2 ± 3.9
Age at disease onset (y)	51.8 ± 11.2	61.9 ± 11.5	48.7 ± 4.9
Gender (male)	20 (71.4%)	3 (50%)	4 (100%)
Duration of disease (y)	4.6 ± 3.8	3.2 ± 1.6	2.5 ± 1
Hoehn and Yahr scale	1.9	–	–

Abbreviations: MSA, multiple system atrophy; PSP, progressive supranuclear palsy.

The mean H&Y stage of IPD patients was 1.9 ( Table 1 ). Most of the included patients were in H&Y stage II ( *n* = 23) followed by stage I ( *n* = 3). One patient was each of H&Y 1.5 and 2.5.

The presenting symptoms in the IPD group were tremors and posture and gait instability, while in the PSP group it was bradykinesia, and in the MSA group, patients presented mainly with complaints of ataxia. Within the IPD group, 22 (78.5%) had tremor-dominant PD and 6 (21.5%) had postural instability and gait disorder. Most of the patients had bilateral disease at the time of the scan. The laterality of disease was defined based on the earlier side involved by the disease. Based on this, out of the 28 IPD patients, 16 (57.1%) had left-sided disease and 12 (42.9%) had right-sided disease.

### 99mTc-TRODAT-1 Imaging Findings

99mTc-TRODAT scan findings were classified on visual and semiquantitative analysis. Visually, all the patients included in the study showed reduced uptake in either one or both basal ganglia. Out of 38 patients, 3 had mildly reduced, 13 moderately reduced, and 22 had severely reduced uptake in the left basal ganglia. On the right side, 2 showed mildly reduced, 12 moderately reduced, and 24 showed severely reduced tracer uptake.

**Fig. 2:**
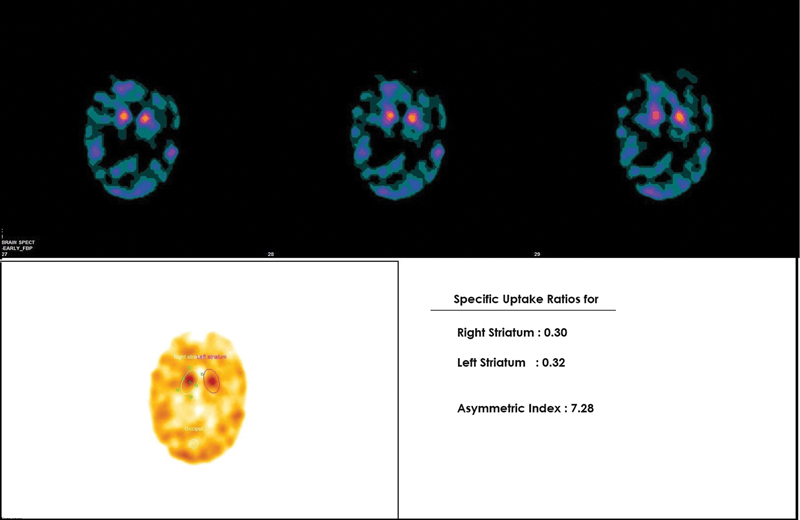
Technetium-99m labeled tropane derivative (99mTc-TRODAT-1) brain single-photon emission computed tomography (SPECT-CT) images of a patient with idiopathic Parkinson's disease (IPD) (Hoehn and Yahr [H&Y] II). Transverse slices show severely reduced uptake in bilateral basal ganglia with more severe reduction in bilateral putamen and relatively preserved uptake in bilateral caudate nucleus, suggestive of reduced presynaptic dopaminergic activity. On semiquantitative evaluation, striatal uptake ratios (SURs) were 0.30 and 0.32 for the right and left striatum, respectively.

The basal ganglia showed inhomogeneous uptake. Thirty-one of 38 patients showed more reduction in uptake in putamen as compared with the caudate nucleus ( Fig. 2 ). Twenty-five of 31 of these patients belonged to the IPD group, 3 to the PSP group, and 3 to the MSA group. There was no significant difference between uptake in IPD, PSP, and MSA subgroups.

On semiquantitative analysis, the mean total, right, and left SUR in the IPD group were 0.58, 0.28, and 0.30 respectively, whereas in the PPS group, it was 0.68, 0.33, and 0.42, respectively ( Table 2 ). The difference in the mean was not statistically significant.

**Table 2:** 99mTc-TRODAT-1 imaging results of the study population

99m Tc-TRODAT imaging	Idiopathic Parkinson's disease ( *n* = 28)	Parkinson-plus syndrome ( *n* = 10)	*p* -Value
Total SUR	0.58 ± 0.27	0.68 ± 0.32	0.34
Right SUR	0.28 ± 0.16	0.33 ± 0.16	0.35
Left SUR	0.30 ± 0.15	0.34 ± 0.17	0.42

Abbreviations: 99mTc-TRODAT-1, technetium-99m labeled tropane derivative; SUR, striatal uptake ratio.

The total (right + left) 99mTc-TRODAT SUR value in the IPD group, PSP group, and MSA group was 0.58 ± 0.27 (0.14–1.26), 0.53 ± 0.31 (0.23–0.96), and 0.91 ± 0.20 (0.73–1.1), respectively ( Table 3 ). In the comparison of IPD versus PPS, no significant difference was observed in the reduction of 99mTc-TRODAT SUR uptake value ( *p* = 0.34). The results showed that the total SUR uptakes were lowest in the PSP group, compared with IPD and MSA, but the difference in SUR values was not statistically significant.

**Table 3:** 99mTc-TRODAT-1 imaging results of the study population

^99m^ Tc-TRODAT imaging	Idiopathic Parkinson's disease ( *n* = 28)	Parkinson-plus syndrome ( *n* = 10)	
		PSP ( *n* = 06)	MSA ( *n* = 04)
Total SUR	0.58 ± 0.27	0.53 ± 0.31	0.91 ± 0.20
Right SUR	0.28 ± 0.16	0.27 ± 0.17	0.43 ± 0.10
Left SUR	0.30 ± 0.15	0.26 ± 0.15	0.47 ± 0.11

Abbreviations: 99mTc-TRODAT-1, technetium-99m labeled tropane derivative; MSA, multiple system atrophy; PSP, progressive supranuclear palsy; SUR, striatal uptake ratio.

**Fig. 3:**
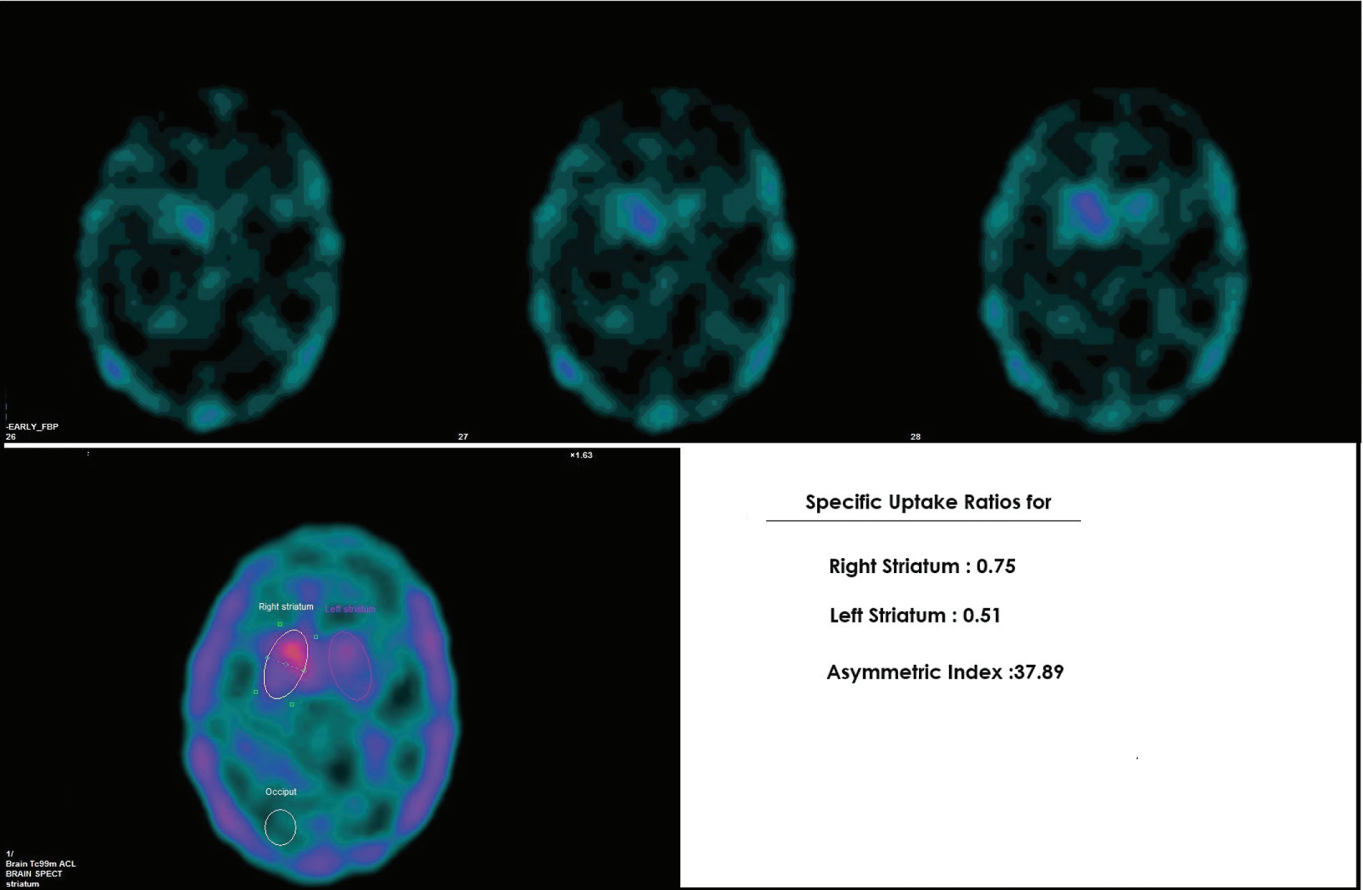
Technetium-99m labeled tropane derivative (99mTc-TRODAT-1) single-photon emission computed tomography (SPECT) images of a patient with idiopathic Parkinson's disease (IPD) (Hoehn and Yahr [H&Y] 1.5). The transverse slices reveal asymmetrically reduced uptake in bilateral basal ganglia with more severe reduction in left putamen and relatively preserved uptake in right caudate nucleus, suggestive of reduced presynaptic dopaminergic activity. On semiquantitative evaluation, striatal uptake ratios (SURs) were 0.75 and 0.51 for the right and left striatum. These findings were concordant with disease lateralization.

Out of 28 patients with IPD, SUR values were reduced in the contralateral striatum (striatum opposite to the side of motor symptoms at disease onset) in 25/28 (89.3%) patients and was consistent ( Fig. 3 ). In the remaining 3/28 (10.7%) patients, a higher reduction in SUR values were observed in the striatum ipsilateral to the side of motor symptoms at the disease onset. Semiquantitatively, SUR values showed a higher reduction in contralateral striatum (contralateral SUR = 0.25 ± 0.14 vs. ipsilateral SUR = 0.32 ± 0.15). The difference was not statistically significant ( *p* = 0.078).

99mTc-TRODAT binding was asymmetric in all the groups of patients. The mean AI in IPD, PSP, and MSA was 43.5 ± 41.6, 33 ± 25, and 12.5 ± 10.3, respectively. Ten of 38 patients had an AI of > 50. Nine of 10 patients among these belonged to the IPD group and the other 1/10 was PSP patient. The majority of the IPD patients were in H&Y stage II ( *n* = 23) followed by stage I ( *n* = 3). Total SUR was found to be inversely related to H&Y stages of IPD ( *p* = 0.241). A similar trend was seen, when contralateral SUR was compared with H&Y stage of IPD ( *p* = 0.065); however, the results were not statistically significant ( Table 4 ).

**Table 4:** Correlation of clinical parameters with the striatal uptake ratio (SUR) in IPD patients ( *n* = 28)

		Age	Age of onset	Duration of disease	Hoehn and Yahr score
Total SUR	rho	–0.056	–0.142	0.131	–0.229
	*p*	0.777	0.470	0.506	0.241
Contralateral SUR	rho	0.016	–0.068	0.277	–0.354
	*p*	0.93	0.732	0.153	0.065
Ipsilateral SUR	rho	–0.108	–0.179	–0.011	–0.021
	*p*	0.586	0.362	0.955	0.917

Abbreviation: IPD, idiopathic Parkinson's disease.

Note: rho: Spearman's correlation coefficient, *p* -value < 0.05 is considered significant.

## Discussion

Movement disorders encompass a broad spectrum of hypokinetic and hyperkinetic neurologic diseases usually associated with an abnormal function in the basal ganglia. The presentation and progression of these diseases is highly variable. 1 The common neurological movement disorders include PD, essential tremor, and dystonia. 1 Many of these disorders have similar clinical presentations, thus making exact diagnosis exceptionally difficult. 10 11 Anatomical imaging with CT and MRI offers limited benefit in diagnosis and differentiation of these movement disorders. Functional imaging with ^99m^ Tc-TRODAT-1 imaging can help in early and accurate diagnosis of PD.

Functional imaging with ^99mTc^ -TRODAT-1 classically shows reduced tracer uptake in basal ganglia with more severe and earlier involvement of putamen 3 12 13 as seen in 25/28 patients in our study cohort. Rest 3/28 patients demonstrated reduction of tracer uptake in both putamen and caudate nucleus, which was probably due to advanced disease, leading to involvement of the entire striatum.

Fallahi et al in their study evaluated the role of 99mTc-TRODAT imaging in differentiation of PD from other movement disorders. The authors concluded that atypical parkinsonism have uniform reduction of tracer in Basal ganglia (BG) (both caudate and putamen) while in PD putamen is predominantly involved. 12 In the present study, symmetric involvement was present only in 50% (3/6) cases in the PSP subgroup and 25% (¼) in the MSA subgroup. Possible explanation can be the limited sample size and advanced disease at presentation in few cases.

To increase the reliability and to validate the results, data was subjected to semiquantitative assessment and SURs were calculated. The total (right + left) 99mTc-TRODAT SURs in the IPD group, PSP group, and MSA group was 0.58, 0.53, and 0.91, respectively. This is in agreement with other studies which have reported reduced SURs in cases of IPD, PSP, and MSA. 3 9 13 14

However, there was no significant difference in the reduction of 99mTc-TRODAT SUR in IPD versus PPS ( *p* = 0.34). The total SUR was lower in the PSP group as compared with other groups. This is in agreement with other studies which have also shown no significant difference between IPD and PPS patients. 3 13 15 Functional imaging using other predopaminergic radiotracers like 18F-DOPA and 123I-ioflupane have also demonstrated similar results. 16 17 18 But Antonini et al have demonstrated that striatal uptake of 123I-ioflupane was significantly lower in the PSP group than in the IPD group. 17

Existing literature suggests that the pathognomic feature of parkinsonism is reduced uptake in the striatum contralateral to the clinically dominant side. 9 19 20 In our study, most of the patients had bilateral disease at the time of presentation. The disease laterality was considered based on the site of onset of symptoms. Note that 89.3% (25/28) IPD patients had more reduced SUR in contralateral striatum. However, the difference was not statistically significant ( *p* -value = 0.078). This might be attributed to advanced disease state among the patients that are included in the current study. Geng et al 21 reported analogous findings in their study, as they noted a similar trend of gradual decline in specific 99mTc-TRODAT uptake in the bilateral striata as IPD progressed from H&Y stage I to III.

In the current study, occasional ipsilateral reduction of SUR was observed in 10.7% (3/28) of patients. This phenomenon can be attributed to the fact that both the ipsilateral and the contralateral basal ganglia may be associated with abnormal signaling to the cerebello-thalamo-cortical circuit, which is involved in the pathogenesis of several types of tremors. Similar findings have also been reported by Birman et al 22 in their study, where 5/11 patients with ipsilateral striatal involvement had PD on follow-up.

AI is a quantitative parameter to define the asymmetry of SUR between contralateral and ipsilateral striata. Weng et al 9 in their study found that mean AI of the PD group was 65.6, 30.7, and 11.2 in the putamen, whole striatum, and caudate nucleus, respectively. They found that the PD group had significantly asymmetric 99mTc-TRODAT-1 binding ratios. In the current study, mean AI in the IPD group was 43.5 ± 41.6. Ten of 38 patients had an AI of > 50. Nine of 10 patients among these belonged to the IPD group and the other 1 was PSP patient.

Existing literature suggests a restricted utility of presynaptic dopaminergic ligands in distinguishing between IPD and PPS, as both conditions exhibit reduced presynaptic dopaminergic tracer accumulation. 3 13 15 16 17 18 23 The current study aligns with these findings, as it did not reveal any strong, discernible tracer reduction pattern in the striatum that could distinguish between PPS and PD. Available literature also suggests that metabolic imaging with 18F-fludeoxyglucose PET/CT can differentiate IPD from PPS. In IPD, there will be increased metabolic activity in the basal ganglia, thalamus, and pons along with reduced metabolic activity in the parieto-occipital cortices. Whereas PSP will show decrease in metabolic activity in the anterior cingulate cortex, basal ganglia, and midbrain, and MSA will show reduced metabolic activity in the basal ganglia and cerebellum. 24 25

Limitations of our study included small sample size, fewer cases of PPS (including PSP and MSA) than PD, and advanced disease states. Also, no healthy controls were included for comparison and only a single 99mTc-TRODAT-1 SPECT/CT modality was used to evaluate the study population. Thus, a longitudinal study may help characterize and correlate the 99mTc-TRODAT findings with progression of the disease in IPD and PPS patients.

## Conclusion

99mTc-TRODAT is a potential imaging biomarker for the evaluation of presynaptic dopaminergic dysfunction. The current study evaluated the role of 99mTc-TRODAT in the evaluation of movement disorders and differentiation of PPS and IPD. In our study cohort, mean SUR values were lowest for the PSP group followed by the IPD and MSA group, which is in concordance with previous studies. However, the difference between SUR values of two groups (IPD vs. PPS) was not statistically significant. The present study emphasizes that the ability of 99mTc-TRODAT imaging alone for diagnosing IPD from PPS is constrained, although it offers a precise approach for distinguishing patients with IPD from those with essential tremors, drug-induced, or psychogenic parkinsonism. Consequently, more specific imaging biomarkers are required to effectively differentiate between patients with IPD and those with PPS.
